# Neuroinflammation of microglia polarization in intracerebral hemorrhage and its potential targets for intervention

**DOI:** 10.3389/fnmol.2022.1013706

**Published:** 2022-10-11

**Authors:** Guoqiang Yang, Xuehui Fan, Maryam Mazhar, Wubin Guo, Yuanxia Zou, Nathupakorn Dechsupa, Li Wang

**Affiliations:** ^1^Research Center for Integrated Chinese and Western Medicine, The Affiliated Traditional Chinese Medicine Hospital of Southwest Medical University, Luzhou, China; ^2^Molecular Imaging and Therapy Research Unit, Department of Radiologic Technology, Faculty of Associated Medical Sciences, Chiang Mai University, Chiang Mai, Thailand; ^3^Acupuncture and Rehabilitation Department, The Affiliated Traditional Chinese Medicine Hospital of Southwest Medical University, Luzhou, China; ^4^Key Laboratory of Medical Electrophysiology, Ministry of Education and Medical Electrophysiological Key Laboratory of Sichuan Province, Collaborative Innovation Center for Prevention of Cardiovascular Diseases, Institute of Cardiovascular Research, Southwest Medical University, Luzhou, China; ^5^First Department of Medicine, Medical Faculty Mannheim, University Medical Centre Mannheim (UMM), University of Heidelberg, Mannheim, Germany; ^6^National Traditional Chinese Medicine Clinical Research Base and Drug Research Center of the Affiliated Traditional Chinese Medicine Hospital of Southwest Medical University, Luzhou, China; ^7^Institute of Integrated Chinese and Western Medicine, Southwest Medical University, Luzhou, China; ^8^Department of General Surgery, The Affiliated Traditional Chinese Medicine Hospital of Southwest Medical University, Luzhou, China

**Keywords:** intracerebral hemorrhage, microglia, phenotype shift, neuroimmunology, neuroinflammation

## Abstract

Microglia are the resident immune cells of the central nervous system (CNS) and play a key role in neurological diseases, including intracerebral hemorrhage (ICH). Microglia are activated to acquire either pro-inflammatory or anti-inflammatory phenotypes. After the onset of ICH, pro-inflammatory mediators produced by microglia at the early stages serve as a crucial character in neuroinflammation. Conversely, switching the microglial shift to an anti-inflammatory phenotype could alleviate inflammatory response and incite recovery. This review will elucidate the dynamic profiles of microglia phenotypes and their available shift following ICH. This study can facilitate an understanding of the self-regulatory functions of the immune system involving the shift of microglia phenotypes in ICH. Moreover, suggestions for future preclinical and clinical research and potential intervention strategies are discussed.

## Introduction

Intracerebral hemorrhage (ICH) is a destructive disease because of its increased mortality and morbidity rates, accounting for nearly 10%–20% of stroke cases worldwide (Dasari et al., 2021; Liu et al., 2022; Yang et al., 2022). After ICH, vessel rupture of brain parenchyma contributes to the aggregation of red blood cells (RBCs) and the formation of hematoma to oppress brain tissue structure forming primary brain injury (PBI). Erythrocyte hemolysis in hematoma results in secondary brain injury (SBI) and non-reversing neurological deficits duet o the toxic hemolytic products (Ziai, 2013). Surgical treatment is not conducive to a hematoma in the majority of hemorrhage strokes due to doubtful clinical effectiveness and side effects of surgery (Hemphill et al., 2015). Evidence suggests that inflammatory responses firmly participate in and contribute to the SBI pathophysiological processes following ICH (Chen S. et al., 2015). During this pathological process, the CNS resident microglia and monocytes derived macrophages infiltrate from the circulation at the hemorrhagic site. These microglia/macrophages act as primary modulator for the hematoma resolution and alleviation of neuroinflammation in SBI (Bai et al., 2020; Liu et al., 2022).

Microglia, as the primary immune cells, account for 5% to 10% of brain cells in the central nervous system (CNS) and are referred to as the brain’s macrophage (Eldahshan et al., 2019; Bian et al., 2020). Following physiological conditions, microglia interact with other cells, including neurons, astrocytes, and oligodendrocytes, displaying their function in the brain following ICH (Prinz et al., 2019). It is essential in sustaining brain homeostasis. When different neuropathologic changes disrupt normal function in the brain, activated microglia exert their regulatory effects (Wan et al., 2016). The highly diverse property of microglia and their phenotypes depend on the kinds of stressors or neuropathology (Wolf et al., 2017). Specifically, microglia polarization produces pro-inflammatory (IL-1β, IL-6, TNF-α, CXCL8, CCL2, and CCL5) or anti-inflammatory (IL-4, IL-10, IL-13, IL-1Ra, and TGFβ) mediators during the different pathological phases, participating in ICH progression (Friedman et al., 2018; Bai et al., 2020).

Pathological analyses following ICH have uncovered that microglia mediated neuroinflammation can be recognized as a major contributor to inflammatory injury following ICH (Yang et al., 2014a; Zhang Z. et al., 2017). The severe microglial neuroinflammation caused by the hematoma and hemolysis after ICH contributes to brain injury (Vinukonda et al., 2019; Wang et al., 2019a). Chang et al. (2017) have proven that microglia quickly responded to hemorrhagic damage after 1–1.5 h of ICH onset and showed a microglial protective phenotype. In this study, IL-10 mediated microglia phagocytosis and hematoma resolution (Chang et al., 2017).

Different microglial phenotypes play a primary and complex function in the SBI-induced inflammatory damage and brain rehabilitation following ICH. Therefore, the neuroprotective effect of microglia can be regarded as a hopeful target in the inflammatory response following ICH therapy. Jing et al. (2019) have reported that after blocking the erythrocyte CD47, activated microglia can be enhanced to phagocytose the hematoma and reduce neurological deficits, brain edema, and neuronal reduction (Chang et al., 2018). Microglial depletion contributes to intense brain tissue damage, including brain swelling, neuronal loss, and neurological defects following ICH (Jin et al., 2017; Sekerdag et al., 2018; Yang X. et al., 2018). The reactive microglia show a biphasic influence on responding to inflammation processes following ICH, which acts as a double-edged sword displaying offensive and defensive effects in brain injury. This study focuses on current empirical investigations on reactive microglia in the ICH-induced SBI pathological process to elucidate how the pivotal elements affect this process, providing an optimistic viewpoint for improving novel medicinal strategies.

## Microglial Function Following ICH

ICH-induced hematoma is a significant factor resulting in brain damage following ICH. The compression and dissection of mechanical impairment obstruct adjacent brain parenchyma structures. Simultaneously, within hours to days, extravasated erythrocytes release blood products with neurotoxicity, including cytotoxic hemoglobin, heme, and iron in the hematoma, which initiate SBI inducing sustained cerebral edema and brain tissue damage following ICH (Zhao et al., 2009; Wang G. et al., 2018). This process can be regulated through the activation of peroxisome proliferator-activated receptor γ (PPAR-γ) and modulation of the CCR4/ERK/Nrf2 signaling pathway to promote phagocytosis and shift microglia for conferring immune balance (Deng et al., 2020; Tschoe et al., 2020; Zhuang et al., 2021).

The collapse of the blood-brain barrier (BBB) and its resulting brain swelling is a notable life-threatening event in the pathophysiology of hemorrhagic stroke (Su et al., 2017; Fang et al., 2020). Ample evidence has shown that it is essential that microglial activation displays a significant role in SBI after ICH (Carson et al., 2006). It has been reported that M1 polarization of microglia provokes the production of pro-inflammatory factors, such as TNF-α, and IL-6, which aggravate the inflammatory response (Wang, 2010; Zhou et al., 2014). Chen A. Q. et al. (2019) have shown that M1-type microglia-induced TNF-α mediates endothelial necroptosis leading to BBB disruption. After being given the anti-TNF-α treatment, pathologic changes, including endothelial necroptosis and BBB destruction, and stroke outcomes were significantly alleviated following ischemic stroke (Chen A. Q. et al., 2019). Besides, some medicines displaying neuroprotective function attenuated the LPS-induced NO release, TNF-α secretion, and NFκB expression of microglia to mediate BBB protection and neural repairation by promoting the production of anti-inflammatory cytokines (Zlokovic, 2008; Liu et al., 2017; Chen J. et al., 2019). Similarly, IL-4 and IL-10 induced an alternative microglia phenotype with anti-inflammatory function, which can be recognized as therapeutic targets to modulate the BBB physiology in ICH (Ronaldson and Davis, 2020).

### Microglia phenotypes and their polarization after ICH

Previously, microglia polarization associated with M1 and M2 phenotypes was widely used in previous research work. Due to the development of omics technology in experiments, recently, a novel class of microglia polarization bridges the standard M1/M2 contradiction, resulting in an intense controversy to further study microglia polarization (Ransohoff, 2016).

First, Chiu et al. (2013) utilized flow cytometry and deep RNA sequencing to indicate that microglia isolated from the SOD1 (G93A) mutant mouse model of amyotrophic lateral sclerosis (ALS) differed from SOD1 (WT), LPS-induced microglia, and M1/M2 macrophages. This study freshly provided a new definition for ALS-specific microglia’ functional phenotypes in isolated spinal cord microglia of ALS mice model (Chiu et al., 2013). Besides, single-cell RNA-sequence (scRNA-seq) analyses have suggested assembled expression of M1 and M2 markers and complex microglia-state changes in the total mouse lifespan and the damaged brain (Ajami et al., 2018; Hammond et al., 2019; Masuda et al., 2019). Furthermore, accurate classification of different microglia subpopulations is founded upon species-related differences and different pathological environments. Different subtypes of microglia are associated with a core pattern of activation, in which the pro-inflammatory-activation state shifts toward an anti-inflammatory situation in maintaining tissue homeostasis or resulting in CNS pathology (Voet et al., 2019; Lassmann, 2020). However, due to the complicated existence of microglial phenotypes found by novel technologies in diseases, the established meaningful program of microglial polarization of M1 and M2 phenotypes hindered research progress and should need to be debated (Ransohoff, 2016). Overall, an elusive definition of microglial polarization exists, and it is oversimple to describe M1 or M2 phenotypes for the complex biology of microglia.

Recently, a study performed by Keren-Shaul et al. (2017) specified a subtype of microglia named disease-associated microglia (DAM), which develops in two steps in Alzheimer’s disease (AD)-transgenic (Tg-AD) and triggering receptor expressed on myeloid cells 2 (TREM2)^–/–^ Tg-AD mouse brains by transcriptional single-cell analysis (Da Mesquita and Kipnis, 2017; Ozaki et al., 2022). Microglial activation to DAM is instigated in an independent program away from TREM2, followed by activation in a TREM2-dependent manner. Such a special microglial type has the prospect of confining neurodegeneration, which may have meaningful implications for future therapy of AD and other neurodegenerative illnesses (Keren-Shaul et al., 2017; Deczkowska et al., 2018). Subsequently, genome-wide transcriptomic analyses (GWTAs) indicated DAM’s existence under various pathological disorders, including aging and ALS pathology (Keren-Shaul et al., 2017). Although the microglia DAM and M1 phenotype gene profiles are partially overlaid, the apparent differences in their molecular signatures exist (Garcia-Revilla et al., 2019). Interestingly, a transcriptomic framework of microglial activation uncovered that DAM demonstrates double anti-inflammatory and pro-inflammatory sub-profiles in (AD and aging models; Rangaraju et al., 2018; Gao et al., 2019). The progressive transition from homeostatic microglia to reactive DAM relies on TREM2, which is mainly located on the microglia surface in brain tissue (Arcuri et al., 2017; Mecca et al., 2018; Xu et al., 2022). The activated TREM2 alleviated microglia neuroinflammation, neurological deficits, and neuronal loss by activating the PI3K/Akt signaling pathway in perihematomal areas following ICH (Chen et al., 2020).

Moreover, in 2019, Gao et al. (2019) reported that the regulators, including CEBPα, IRF1, and LXRβ, modulate pro- and anti-inflammatory DAM genes *via* Erk signaling. However, some researchers reported that DAM represents a switch depending on TREM2, a risk gene, and such a switch substantially alters microglial function (Brown and St George-Hyslop, 2017). Therefore, it is pretty clear that microglia is exquisitely sensitive to brain tissue pathological changes.

Multiple investigations have explored microglial spatiotemporal fixed subclasses during evolution and illness, determining the precise molecular hallmarks and various cellular kinetics utilizing single-cell analyses (Prinz et al., 2017; Masuda et al., 2019). Olah et al. (2020) have suggested the existence of four microglial subclasses and clarified the importance of these subclasses according to the scRNA-seq from the human cerebral cortex samples associated with AD. Lately, a study by Ochocka and his colleagues has demonstrated microglia cellular and functional heterogeneity utilizing scRNA-seq and flow cytometry. Their investigation obtained numerous microglial groups and gene expression profiles underlying a distinct group image with different roles. Reactive microglia displayed the distinct spatial distribution in naïve and GL261 glioma-bearing mice through performing the scRNA-seq of CD11b^+^ myeloid cells (Ochocka et al., 2021). Furthermore, the technologies of transcriptomes and epigenetic landscapes have been utilized by Gosselin et al. (2017) to examine separated human microglia derived from surgically resected brain tissue, uncovering an environment-dependent transcriptional network that can sufficiently specify microglia-specific schedules of gene expression. These results can identify significant microglia-subclasses associated with neurodegenerative diseases and behavioral disorders and can be used to understand microglia’s roles in human brain diseases (Gosselin et al., 2017).

**Figure 1:**
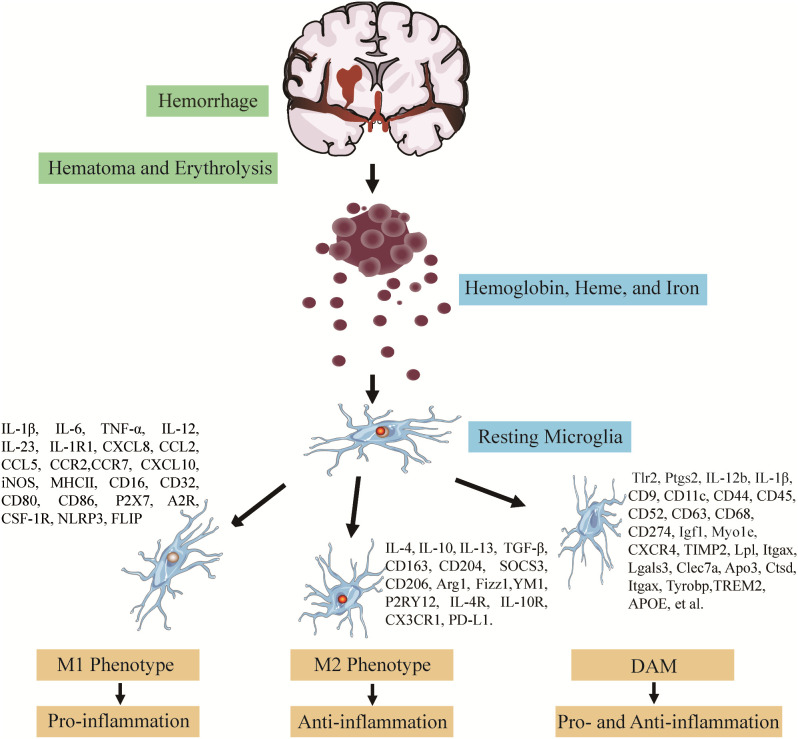
Polarization of activated microglia following intracerebral hemorrhage (ICH). The polarization of activated microglia in response to stimulation with erythrocyte lysates after ICH can be broadly classified into three categories. Each category has its related cytokines, chemokines, and surface markers: (1) pro-inflammatory microglia elevate the pro-inflammatory factors and destruc­ tive effects on the brain; (2) anti-inflammatory microglia mainly exert neuroprotective effects; (3) disease-associated microglia (DAM). Recent, an increasing number of studies by single-cell RNA-sequence and Various other omics methods have revealed that microglia can also be polarized into neuroprotective and neurodestructive phenotypes, remaining to be further investigated.

Diverse pathologic occurrences or changes in the brain activate microglia, whose intricate functions facilitate the presence of individual pro- and anti-inflammatory effects in ICH. The meaning of defining microglial subclasses through single-cell analysis is to confirm precise microglia function and identify microglia-subclasses molecules mediating astrocyte-microglial communication. In addition, the importance of clustering negotiate its potentially coordinated functions with astrocytes and discover new ways to address the complexity of biology in inflammatory conditions and different pathologies (Vainchtein and Molofsky, 2020). During the ICH progress, microglial activation and polarization modulators have clinical and translational implications, including critical signaling pathways, transcription factors (TFs), and particularly microglia M1- and M2-subclasses markers, providing evidence for coordinating microglial function to alleviate ICH-induced brain damage (Lan et al., 2017). The pro-inflammatory microglia-induced damage-enhancing mediators, such as inflammatory cytokines, chemokines, matrix metalloproteinases, and reactive oxygen species, overwhelmed the anti-inflammatory microglia with potential reparative roles after the onset of ICH (Bai et al., 2020). Therefore, confirming the modulators and these influencing factors makes it attractive to induce the microglia polarization to a neuroprotective subclass, which provides novel insight into alleviating the microglia pathologic changes after ICH. According to the above analyses of microglia polarization in ICH and studies about surface markers of microglial subtypes (Klebe et al., 2015; Keren-Shaul et al., 2017; Rangaraju et al., 2018; Bai et al., 2020), it is critical to shift the microglia pro-inflammatory to the anti-inflammatory subclass to improve the outcomes following SBI (Figure 1).

## Potential Therapeutic Targets and Strategies of Microglia-Induced Neuroinflammation After ICH

Many studies have emphasized the autogenous regulation of microglia during ICH progress. Wu et al. (2017) have shown that the expression of soluble epoxide hydrolase increased in microglia following ICH, resulting in neuroinflammatory responses, and inhibiting its expression can suppress microglia-mediated neuroinflammation. The deficiency of TWIK-related K^+^ channel 1 (TREK-1) can induce increased recruitment of microglia and neutrophils and the production of pro-inflammatory factors following ICH-induced SBI; the TREK-1 can be harnessed into a promising therapeutical target in BBB dysfunction and microglia neuroinflammation for the treatment of ICH (Fang et al., 2019). Besides, the integrin Mac-1 expressed by microglia acting together with the endocytic receptor LRP1 in the neurovascular unit promoted thrombolytic tissue plasminogen activator (tPA)-induced platelet-derived growth factor-cc (PDGF-cc) activation, which increased the permeability of BBB following ischemic stroke (Su et al., 2017).

On the contrary, microglia are implicated in anti-inflammatory and phagocytic effects to improve neurologic deficits following ICH. The relationship between regulatory T lymphocytes (Tregs) and microglia neuroinflammatory reaction has been verified. A study by Zhou et al. (2017) demonstrated that Tregs alleviated ICH-mediated neuroinflammation because of the shift of the M2 anti-inflammatory from the M1 pro-inflammatory subclass *via* the regulation of the IL-10/GSK3β/PTEN axis after ICH (Wang et al., 2015; Taylor et al., 2017).

### Regulatory mechanisms in microglia-induced neuroinflammation in ICH

After ICH, the functions of anti-inflammatory microglia are performed through diverse signal axes that constitute a complicated network implicated in numerous biological procedures. Investigating the related biological signaling pathways and their molecular foundation is beneficial to illustrating attractive methods to moderate targets, thus improving the neuropathological deficits in ICH-induced brain damage.

Some studies have verified that adenosine monophosphate-activated protein kinase (AMPK) regulated the balance for the switch between a pro- and an anti-inflammatory subclass, which serves as a primary sensor of brain injuries and diseases, and is considered a candidate molecule (Ohnishi et al., 2007; Saito et al., 2019). Adiponectin receptor 1 (AdipoR1) is always expressed by microglia, and the expression level of endogenous C1q/TNF-related protein 9 (CTRP9), activating AdipoR1 to regulate the AMPK signaling pathway, plays an increasing trend during the first 24 h after ICH (Zhao et al., 2018). Administration of CTRP9 treatment improved AdipoR1 and p-AMPK protein expression levels and decreased the protein expression levels of inflammatory cytokines and phosphorylated NFκB (P-NFκB), attenuating neuroinflammation *via* AdipoR1/AMPK/NFκB signaling pathway (Zhao et al., 2018). Activated AdipoR1 by CTRP9 treatment attenuated neuropathological deficits and improved BBB dysfunction by activating the APPL1/AMPK/Nrf2 signal axis in a collagenase-induced ICH mouse model (Xu et al., 2018; Zhao et al., 2021). This evidence suggested that CTRP9 could be recognized as an encouraging therapy to ameliorate BBB dysfunction in ICH patients. Likewise, the activation of melanocortin receptor 4 (MC4R) improves neuropathological function *via* the AMPK signal, and intervening MC4R can be effective in animal experiments and utilized as a potential therapeutic approach for ICH management (Chen et al., 2018).

As mentioned above, Treg cells restrain microglia-mediated neuroinflammation from improving neurological function by activating NFκB through the JNK/ERK pathway (Yang et al., 2014b; Lan et al., 2017; Zhou et al., 2017). The treatment of hyperbaric oxygen preconditioning (HBOP) has attenuated the production of pro-inflammatory cytokine levels and p-JNK, suggesting potential relevance between JNK phosphorylation and downregulation of immunoactivity and protein levels of M1 markers (Yang L. et al., 2015; Wang et al., 2019b). As many limitations, including hesitations concerning effectiveness, surgical damages, complications, and drug side effects, exist in current clinical practice, there are few standardized clinical interventions for ICH treatment. Therefore, hyperbaric oxygen therapy provides a potential alternative medicine to treat ICH, and the mechanisms of HBOP for intervening in ICH need additional investigation and confirmation.

Toll-like receptor 4 (TLR4) confer an essential function in the innate immune response, known as a pattern recognition receptor (Fang et al., 2013). The deficiency of TLR4 attenuated perihematomal inflammatory response associated with a decrease in the recruitment of pro-inflammatory microglia in a striatal blood injection-induced ICH mice model (Sansing et al., 2011; Wang et al., 2013). Moreover, TLR4 also inhibits the microglial phagocytic capacity for RBCs, contributing to the deceleration of CD36-mediated hematoma absorption and severe neurological deficits in ICH (Fang et al., 2014; Li Q. et al., 2021). Autophagy mediated by TLR4 activated microglia-induced neuroinflammation in mice with ICH (Yang Z. et al., 2015). The function of TLR4 has been elaborated in detail following ICH in many studies. Therefore, therapeutic strategies targeting TLR4 are relatively promising interventions and may represent future candidates for ICH therapy.

DNA damage motivates the body’s innate immune response. As a DNA sensor, cGMP-AMP synthase (cGAS) can detect the disease-damaged DNA and triggers its downstream stimulator of interferon gene (STING), subsequently phosphorylating interferon regulatory factor 3 (IRF3) to upregulate the production of type I interferon (IFN; Lei et al., 2022). cGAS is a critical regulator of inflammatory and autophagy responses, and Sharma et al. (2020) found that cGAS is upregulated to mediate inflammation through increasing inflammatory genes (Ccl5 and Cxcl10) and autophagy responses *via* activating the two major autophagy initiators, LC3A and LC3B, in striatal damage of brain. It has been reported that tPA administration augments neutrophil extracellular traps (NETs) markers in the ischemic mice brain cortex and their plasma. DNase I and deficiency of peptidyl arginine deiminase 4 (PAD4), which can inhibit NETs, reversed tPA-mediated upregulation of cGAS.However, cGAMP application suppressed DNase I-mediated antihemorrhagic effects by downregulating the STING and INF in tPA-treated mice following ischemic stroke (Wang et al., 2021). Moreover, Jiang et al. (2021) have elucidated that cGAS knockdown improved M2 phenotype polarization of microglia to attenuate microglial inflammatory response by hindering the cGAS-STING signal axis in mice with stroke, highlighting that such signal axis can be used as a potential therapeutic target. Subsequently, the experiment performed by Shi et al. (2022) also demonstrated that inhibiting the cGAS-STING pathway through a cGAS inhibitor integrated versatile immunosuppressive nanoparticle in microglia contributed to improving an anti-inflammatory phenotype polarization in rats following stroke.

It is well known that c-type lectin-like receptors (CLRs) are mainly expressed in myeloid cells as a family of transmembrane pattern recognition receptors (Drouin et al., 2020). CLRs’ dysregulation results in the production of inflammatory mediators and the development of inflammatory diseases following excessive injury. Microglial macrophage-inducible C-type lectin (Mincle), a critical member in CLRs, widely expressed on antigen-presenting cells (APCs), including macrophages, binds nuclear spliceosome-Associated Protein 130 (SAP130) from necrotic cells to enhance neuroinflammation (Del Fresno et al., 2020; He et al., 2022). After the injury, Mincle and its activated downstream spleen tyrosine kinase (Syk) boost inflammatory gene expression levels in alcohol-induced liver injury mice (Kim et al., 2018). Besides, activated Mincle/Syk signal worsened intestinal mucosal inflammation by enhancing macrophage pyroptosis, and inhibition of the Mincle/Syk signaling pathway displayed a potential therapeutic function to attenuate inflammatory response in Crohn’s Disease (Gong et al., 2020). Furthermore, many investigations have suggested that inhibiting the Mincle/Syk signal axis exerts a neuroprotective role associated with various brain diseases in preclinical research. Different intervention treatments, including Syk inhibitor BAY61-3606, acupuncture, and MSCs engraftment, have been used to attenuate microglia-mediated neuroinflammation by impeding Mincle/Syk signaling pathway in microglia following hemorrhage stroke, ischemic stroke, and traumatic brain injury (TBI; He et al., 2015, 2022; de Rivero Vaccari et al., 2015; Liu X. Y. et al., 2018; Li Y. et al., 2021). According to the above analysis, the Mincle/Syk signaling pathway can be utilized as a promising therapeutical target in ICH.

**Figure 2:**
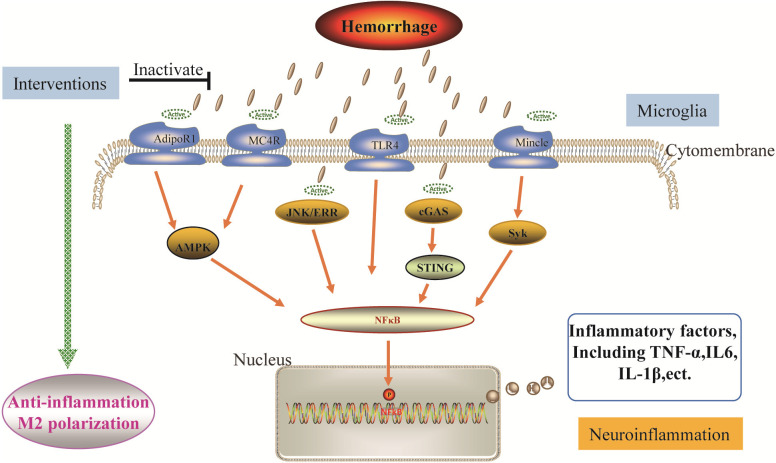
Potential interventions for the neuroinflammation following ICH. Interventions induce the polarization of pro-inflammatory microglia towards the anti­ inflammatory phenotype through different mechanisms, exerting beneficial effects. The mechanisms associated with ICH include the AdipoR1/MC4R-AMPK pathway, JNK-ERR pathway, TLR4 pathway, cGAS-STING pathway, and Mincle-Syk pathway, activation of which enhances the microglial neuroinflammation. After the corresponding intervention inhibits these pathways, the Ml phenotype shifts to the M2 phenotype of microglia.

Although much pre-clinical research has proved the mechanisms against microglial neuroinflammation, there is still a need for further investigation of promising interventions to promote its use in clinical research (Figure 2).

### Therapeutic targets and strategies for microglia-induced neuroinflammation in ICH

Increasing genetic and epigenetic evidence has demonstrated that miRNAs confer crucial functions in regulating gene expression and microglia polarization after ICH (Yang Z. et al., 2018). Yang Z. et al. (2018) found that let-7a regulates microglia M2 polarization at 3 days after ICH in mice by intervening in a target gene named Casein Kinase 2 Interacting Protein 1 (CKIP-1). In this study, overexpressing let-7a lessened the protein expression level of CKIP-1, enhancing microglia M2 polarization (IL-10 and Arg-1) and alleviating the inflammatory response, while inhibiting let-7a augmented the protein expression level of CKIP-1, contributing to microglia M1 polarization (IL-1β and TNF-α). Overexpression of miRNA-7 can restrain TLR4 protein expression level from alleviating the microglia inflammatory response in ICH rats and a lipoprotein-induced microglial inflammation model (Zhang et al., 2018). Further studies have suggested that targeting TLR4 exerted a neuroprotective role in resisting ICH-induced brain injury by impeding the Prx1/TLR4/NFκB signaling axis at 3 days after ICH, providing a promising anti-neuroinflammatory approach for hemorrhagic stroke (Liu et al., 2016). Meanwhile, studies have reported that miRNA-182-5p and miRNA-27a modulate the inflammatory response by targeting TLR4 in a middle cerebral artery occlusion (MCAO) rat model and lipopolysaccharide (LPS)-stimulated microglia respectively (Lv et al., 2017; Wang J. et al., 2018). In their studies, overexpression of miRNA-182-5p and miRNA-27a downregulated the protein expression level of TLR4 resulting in the increase of released inflammatory factors.

Recent evidence has clarified that blockage of miRNA-222 attenuated inflammation in erythrocyte lysate-induced microglia and improved brain water content (BWC), neuropathologic deficits, and inflammatory response in ICH mice. In this study, integrin subunit β8 (ITGB8) was specified as a direct target modulated in the negative by miRNA-222, alleviating inflammation and apoptosis in microglia (Bai and Niu, 2020). Previously, the choline of inflammatory response was inhibited by miRNA-132 by intervening in acetylcholinesterase (AChE). A study by Zhang Y. et al. (2017) found that overexpressing miRNA-132 with an injection of lentiviruses encoding miR-132 into the right caudate nuclei before 14 days inhibited the activation of pro-inflammatory microglia, improved BBB dysfunction, and decreased neuronal loss at day 3 in autologous blood-induced ICH. Additionally, miRNAs, the pivotal mediators in autophagic activation-induced inflammation of microglia, can posttranscriptionally and negatively modulate gene expression and function (Wang et al., 2017). Wang et al. (2017) and Yu et al. (2017a) found that miRNA-144 can enhance hemoglobin-mediated activation of the microglial autophagic inflammatory response by directly targeting mTOR’ 3’ untranslated regions (UTRs) to downregulate the gene and protein expression level of mTOR in a hemoglobin-mediated primary hippocampal microglial cell inflammatory model or autologous blood-induced ICH mice after 24 h. Similarly, miR-124 improved microglia M2 phenotype polarization to alleviate inflammatory injury by targeting the 3’-UTR of C/EBP-α *in* *vitro* and *in vivo* experiments (Yu et al., 2017b). The administration of miR-124 mimics significantly alleviated BWC, neurological deficits, C/EBP-α gene, and protein expression levels compared with those in the injection of miR-124 inhibitor in mice with ICH at 3 days. Furthermore, a similar negative regulation of miR-124 to C/EBP-α in gene and protein expression levels was also observed in transduced microglia with miR-124 mimics or miR-124 inhibitors, which were stimulated with erythrocyte lysates.

Up to now, autophagy is a dualistic function, and it is difficult to assess whether it has harmful or beneficial effects after ICH. Excessive autophagy has been reported to worsen endoplasmic reticulum stress (ERS)-mediated brain impairment at 6 h following ICH. However, autophagy strengthened the protective function of ERS by removing the cell debris at 7 days following ICH-induced SBI in rats (Duan et al., 2017). Tan et al. (2020) have elucidated that enhancing autophagy attenuated oxidative stress damage after ICH *via* increasing the expression levels of antioxidant proteins; on the contrary, the autophagy inhibitor reversed the neuroprotection after ICH. Recently, some studies showed that autophagy positively modulates inflammation in ICH (Shi et al., 2018; Xiao et al., 2020).

The interleukins (ILs) levels regarding the ICH advancement are modulated through the intervention of microglial functions. Xu et al. (2020) have found that the treatment of intranasal delivery of IL-4 nanoparticles activating the IL-4/STAT6 axis improved extended functional recuperation and hematoma resolution in collagenase- and blood-induced ICH mice models. On the contrary, IL-15, as a pro-inflammatory cytokine, coordinates the homeostasis and microglia immunoreactive intensity after CNS inflammatory occurrences, and upregulation of the expression level of IL-15 in astrocytes exacerbates brain edema, neurological deficits, and microglia inflammatory factors’ expression by mediating the crosstalk between astrocytes and microglia in patients and mice with ICH (Shi et al., 2020). Besides, Yu et al. (2016) have found that an IL-17A-neutralizing antibody in opposition to IL-17A can attenuate microglial activation and block ICH-induced cytokine expression levels, including TNF-α, IL-1β, and IL-6. The study by Shi et al. (2018) further illustrated that microglial autophagy and neuroinflammation could be boosted by IL-17A; utilization of an IL-17A-neutralizing antibody remarkably diminished brain edema and enhanced neurological deficits in mice with ICH; suppressing ATG5 and ATG7, the essential autophagy genes of autophagy, decreased microglial autophagy and inflammation (Yuan et al., 2017). Another study also found that intraventricular injection of IL-33 ameliorated neuronal and white matter damage-induced neurological dysfunction following ICH by promoting the microglia M2 polarization (Chen Z. et al., 2019).

Considerable evidence suggests that NFκB translocates to the nucleus after ICH, which produced pro-inflammatory factors, including TNF-α and IL-6, which respond to the series of pathological changes. It indicates that inhibiting the NFκB signaling pathway through different interventions, such as miRNAs, GATA-binding protein 4, and some Chinese medicines, provides a more available anti-neuroinflammatory strategy and therapy for ICH treatment (Dong et al., 2011; Hu et al., 2011; Liu et al., 2016; Shang et al., 2019; Xu et al., 2019). Therefore, regulation of NFκB activity confers hopeful clinical usefulness in ICH.

Many studies have proved that the inhibition of glycogen synthase kinase-3β (GSK-3β) exerts a neuroprotective function in animal experiments after ICH (Zhao et al., 2017, 2019; Zheng et al., 2017). The inhibition of GSK-3β significantly improved the hematoma resolution and cognitive deficits through the enhancement of microglia phagocytosis and differentiation of M2-phenotype microglia in rats with ICH (Liu Z. et al., 2018; Li R. et al., 2019). The study by Zhao et al. (2019) demonstrated that 6-bromoindirubin-3’-oxime (BIO), utilized as a typical inhibitor of GSK-3β blocking GSK-3βTyr216 phosphorylation, exerted a protective effect against microglia activation-induced neuroinflammation by increasing the number of anti-inflammatory microglia. Besides, it has been shown that LiCl treatment downregulated GSK-3β to decrease the death of mature oligodendrocytes (OLGs) and enhance the expression of brain-derived neurotrophic factor (BDNF; Li et al., 2020).

It has been shown that activation of PPAR-γ by rosiglitazone protected against BBB damage and ameliorated hemorrhage transformation maybe favor microglial polarization toward anti-inflammatory phenotype (Luo et al., 2006; Li Y. et al., 2019). Phagocytosis is necessary to improve the hematoma resolution, which attenuates hemorrhage-induced toxic effects on surrounding brain parenchyma and may be essential for healing following ICH. Zhao et al. (2007) found that PPAR-γ activators remarkably improved PPAR-γ-regulated gene expression, including CD36 and catalase, whereas diminishing pro-inflammatory gene expression, including TNF-α, IL-1β, MMP-9, and iNOS, and neuronal impairment by activating phagocytosis of microglia. Conversely, such phagocytosis function was particularly impeded through PPAR-γ gene knockdown or anti-CD36 antibody following ICH (Zhao et al., 2007). Besides, PPAR-γ activation is also essential for improving the phagocytic capability of the microglia anti-inflammatory phenotype by CD36 (Zhao et al., 2009). Similarly, an exogenous PPAR-γ activator named 15 (S)-hydroxyeicosatetraenoic acid facilitated functional healing and neuroprotection after ICH (Xu et al., 2017). Founded on a thorough fundamental investigation, PPAR-γ activators have been broadly utilized in clinical therapy. Activated PPAR-γ by simvastatin, improving microglia-mediated erythrocyte phagocytosis, and displaying neuroprotective function, has been verified in ICH patients (Chen et al., 2017; Wang Y. et al., 2018).

Matrix metalloproteinases (MMPs) upregulated following ICH symbolize a universal superfamily of structurally associated zinc-dependent endopeptidases and can lessen the extracellular matrix (EM). Activated microglia are involved in MMPs’ synthesis and secretion (Lattanzi et al., 2020). The influence of MMPs on EM collapse initiated through inflammation is a basis of stroke, which has been reported in many studies (Florczak-Rzepka et al., 2012). Wells et al. (2005) studied the function of MMPs in mice with ICH and found that MMP-12 levels were the most elevated. The following experiments showed that MMP-12 null mice demonstrated substantial neurological recuperation of forelimb and decreased reliance on the ipsilateral forelimb relative to WT mice, and more Iba1 immunostaining positive cells conferred with macrophage morphology were conscripted to the injured site in WT mice. This evidence suggests that MMP-12 is harmful and results in the development of SBI following ICH (Wells et al., 2005). Subsequent studies found that MMP-12 expression in the peri-hematoma decreased when given stem cell therapy and minocycline after ICH. Simultaneously, microglia infiltration-induced inflammatory response decreased (Wasserman and Schlichter, 2007; Liang et al., 2014). Therefore, MMP-induced microglial activation has evolved an underlying intervening target for ICH. Minocycline induces activated M1 microglia into the M2 microglia phenotype (Miao et al., 2018). However, Wasserman et al. (2007) found that although minocycline therapy virtually ameliorates the increase of MMP-12 and TNF-α early, its effectiveness is yielded at 1 week. These results suggested that we should be cautious in inferring ICH from the encouraging effects of minocycline therapy in other brain injury decreases (Wasserman et al., 2007). Other studies also found that stem cell therapy significantly reduced microglial infiltration and MMP-12 expression in surrounding hemorrhage sites following ICH (Liang et al., 2014; Chen M. et al., 2015).

Increasing evidence reveals that the released ferrous iron from erythrolysis is a primary pathogenic factor in hematoma after ICH (Li et al., 2022). The iron toxicity-mediated microglial activation pro-inflammatory response is a substantial reason for brain impairment in ICH. Deferoxamine (DFA) is an iron chelator that can penetrate the BBB and binds to iron. Decreasing iron accumulation through intraperitoneal administration of DFA can moderately promote the outcomes and reduce microglial activation after ICH (Wu et al., 2011; Hatakeyama et al., 2013; Hu et al., 2019). As an inhibitor of microglial activation, minocycline can decrease injured brain iron to prevent neuronal death in ICH (Zhao et al., 2011; Cao et al., 2018). Similarly, as an iron chelator having brain permeability, VK-28 can polarize microglia to a microglial M2 phenotype, reduce BWC, decrease white matter injury, and improve neurobehavioral deficits following ICH (Li et al., 2017; Dai et al., 2019). Observational research proved that it is evident that the complicated regulatory system modulated microglia function, comprehending it critically to clarify phenotypic and genotypic deviations and acquire promising therapies following ICH.

**Figure 3:**
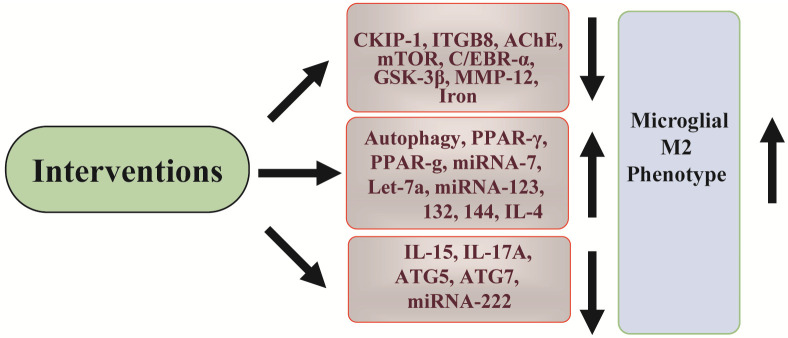
Summary of targets for ICH microglial polarization. Different available interventions can be used to inhibit microglial neuroinflammation. The targets cover signaling pathway proteins, single proteins, genes, and miRNAs. Through modulating these targets, the M1 phenotype of microglia can be switched to the M2 phenotype.

More therapeutic targets have now participated in regulating microglial neuroinflammation; however, further pre-clinical investigation is still needed to promote their use in clinical research (Figure 3).

## Conclusion

This review objectively discusses and assesses the function of microglia activation in modulating ICH-induced brain damage. Increasing evidence suggests that pro- or anti-inflammatory microglia phenotypes have dissimilative functions and meanings, which help us fully comprehend microglia function by regulating related intracellular and extracellular signaling pathways. Moreover, we provide an overall comprehension of cellular and molecular mechanisms responsible for regulating microglia activation after ICH. Although the number of trustworthy clinical trials is reasonably limited and the molecular genetic investigations regarding microglia phenotypic shifts are lacking, our work confers optimistic discernment on a practical intervening approach targeting microglia function in ICH-induced brain injury. Additional studies on therapeutic strategies related to microglia activation-induced neuroinflammation are critical for estimating the possibility of the encouraging treatment noted above.

## Author Contributions

ND and LW: conceptualization, project administration, Science and Technology Project of Sichuan Province, National Natural Science Foundation of China, and funding acquisition. GY and XF: writing—original draft preparation. MM: writing—review and editing. GY, WG, and YZ: visualization. All authors contributed to the article and approved the submitted version.

## Funding

This research was funded by National Traditional Chinese Medicine Inheritance and Innovation Team (No.: ZYYCXTD-C-202207), the Science and Technology Project of Sichuan Province (No.: 2019YFS0543), the Luzhou-Southwest Medical University Science and Technology Strategic Cooperation Project (2021LZXNYD-P04), National Natural Science Foundation of China (No.: 2021XJYJS02), Brain Disease Innovation Team of the Affiliated Traditional Chinese Medicine Hospital of Southwest Medical University (2022-CXTD-05), Luzhou Science and Technology Project (2020, 124), Sichuan Traditional Chinese Medicine Project (2021) No. 13, Southwestern Medical University Hospital (2020) No. 33, and the Project of Southwest Medical University (2021ZKQN125).

## References

[B1] Ajami B. Samusik N. Wieghofer P. Ho P. P. Crotti A. Bjornson Z.. (2018). Single-cell mass cytometry reveals distinct populations of brain myeloid cells in mouse neuroinflammation and neurodegeneration models. Nat. Neurosci. 21, 541–551. 10.1038/s41593-018-0100-x 29507414 PMC8629134

[B2] Arcuri C. Mecca C. Bianchi R. Giambanco I. Donato R. (2017). The pathophysiological role of microglia in dynamic surveillance, phagocytosis and structural remodeling of the developing CNS. Front. Mol. Neurosci. 10:191. 10.3389/fnmol.2017.00191 28674485 PMC5474494

[B4] Bai Y. Y. Niu J. Z. (2020). miR222 regulates brain injury and inflammation following intracerebral hemorrhage by targeting ITGB8. Mol. Med. Rep. 21, 1145–1153. 10.3892/mmr.2019.10903 31894320 PMC7003054

[B3] Bai Q. Xue M. Yong V. W. (2020). Microglia and macrophage phenotypes in intracerebral haemorrhage injury: therapeutic opportunities. Brain 143, 1297–1314. 10.1093/brain/awz393 31919518

[B5] Bian Z. Gong Y. Huang T. Lee C. Z. W. Bian L. Bai Z.. (2020). Deciphering human macrophage development at single-cell resolution. Nature 582, 571–576. 10.1038/s41586-020-2316-7 32499656

[B6] Brown G. C. St George-Hyslop P. H. (2017). Deciphering microglial diversity in Alzheimer’s disease. Science 356, 1123–1124. 10.1126/science.aan7893 28619900

[B7] Cao S. Hua Y. Keep R. F. Chaudhary N. Xi G. (2018). Minocycline effects on intracerebral hemorrhage-induced iron overload in aged rats: brain iron quantification with magnetic resonance imaging. Stroke 49, 995–1002. 10.1161/STROKEAHA.117.019860 29511126 PMC5871578

[B8] Carson M. J. Doose J. M. Melchior B. Schmid C. D. Ploix C. C. (2006). CNS immune privilege: hiding in plain sight. Immunol. Rev. 213, 48–65. 10.1111/j.1600-065X.2006.00441.x 16972896 PMC2633103

[B9] Chang C. F. Goods B. A. Askenase M. H. Hammond M. D. Renfroe S. C. Steinschneider A. F.. (2018). Erythrocyte efferocytosis modulates macrophages towards recovery after intracerebral hemorrhage. J. Clin. Invest. 128, 607–624. 10.1172/JCI95612 29251628 PMC5785262

[B10] Chang C. F. Wan J. Li Q. Renfroe S. C. Heller N. M. Wang J.. (2017). Alternative activation-skewed microglia/macrophages promote hematoma resolution in experimental intracerebral hemorrhage. Neurobiol. Dis. 103, 54–69. 10.1016/j.nbd.2017.03.016 28365213 PMC5540140

[B11] Chen A. Q. Fang Z. Chen X. L. Yang S. Zhou Y. F. Mao L.. (2019). Microglia-derived TNF-α mediates endothelial necroptosis aggravating blood brain-barrier disruption after ischemic stroke. Cell Death Dis. 10:487. 10.1038/s41419-019-1716-9 31221990 PMC6586814

[B12] Chen J. Jin H. Xu H. Peng Y. Jie L. Xu D.. (2019). the neuroprotective effects of Necrostatin-1 on subarachnoid hemorrhage in rats are possibly mediated by preventing blood-brain barrier disruption and RIP3-mediated necroptosis. Cell Transplant. 28, 1358–1372. 10.1177/0963689719867285 31370690 PMC6802141

[B13] Chen M. Li X. Zhang X. He X. Lai L. Liu Y.. (2015). The inhibitory effect of mesenchymal stem cell on blood-brain barrier disruption following intracerebral hemorrhage in rats: contribution of TSG-6. J. Neuroinflammation 12:61. 10.1186/s12974-015-0284-x 25890011 PMC4392640

[B15] Chen S. Peng J. Sherchan P. Ma Y. Xiang S. Yan F.. (2020). TREM2 activation attenuates neuroinflammation and neuronal apoptosis via PI3K/Akt pathway after intracerebral hemorrhage in mice. J. Neuroinflammation 17:168. 10.1186/s12974-020-01853-x 32466767 PMC7257134

[B14] Chen Q. Shi X. Tan Q. Feng Z. Wang Y. Yuan Q.. (2017). Simvastatin promotes hematoma absorption and reduces hydrocephalus following intraventricular hemorrhage in part by upregulating CD36. Transl. Stroke Res. 8, 362–373. 10.1007/s12975-017-0521-y 28102508

[B16] Chen S. Yang Q. Chen G. Zhang J. H. (2015). An update on inflammation in the acute phase of intracerebral hemorrhage. Transl. Stroke Res. 6, 4–8. 10.1007/s12975-014-0384-4 25533878

[B18] Chen Z. Xu N. Dai X. Zhao C. Wu X. Shankar S.. (2019). Interleukin-33 reduces neuronal damage and white matter injury via selective microglia M2 polarization after intracerebral hemorrhage in rats. Brain Res. Bull. 150, 127–135. 10.1016/j.brainresbull.2019.05.016 31129170

[B17] Chen S. Zhao L. Sherchan P. Ding Y. Yu J. Nowrangi D.. (2018). Activation of melanocortin receptor 4 with RO27–3225 attenuates neuroinflammation through AMPK/JNK/p38 MAPK pathway after intracerebral hemorrhage in mice. J. Neuroinflammation 15:106. 10.1186/s12974-018-1140-6 29642894 PMC5896146

[B19] Chiu I. M. Morimoto E. T. Goodarzi H. Liao J. T. O’Keeffe S. Phatnani H. P.. (2013). A neurodegeneration-specific gene-expression signature of acutely isolated microglia from an amyotrophic lateral sclerosis mouse model. Cell Rep. 4, 385–401. 10.1016/j.celrep.2013.06.018 23850290 PMC4272581

[B20] Da Mesquita S. Kipnis J. (2017). DAMed in (Trem) 2 Steps. Cell 169, 1172–1174. 10.1016/j.cell.2017.05.039 28622503

[B21] Dai S. Hua Y. Keep R. F. Novakovic N. Fei Z. Xi G. (2019). Minocycline attenuates brain injury and iron overload after intracerebral hemorrhage in aged female rats. Neurobiol. Dis. 126, 76–84. 10.1016/j.nbd.2018.06.001 29879529

[B22] Dasari R. Bonsack F. Sukumari-Ramesh S. (2021). Brain injury and repair after intracerebral hemorrhage: the role of microglia and brain-infiltrating macrophages. Neurochem. Int. 142:104923. 10.1016/j.neuint.2020.104923 33248206 PMC7818651

[B23] de Rivero Vaccari J. C. Brand F. J. 3rd Berti A. F. Alonso O. F. Bullock M. R. de Rivero Vaccari J. P. (2015). Mincle signaling in the innate immune response after traumatic brain injury. J. Neurotrauma 32, 228–236. 10.1089/neu.2014.3436 25111533

[B24] Deczkowska A. Keren-Shaul H. Weiner A. Colonna M. Schwartz M. Amit I. (2018). Disease-associated microglia: a universal immune sensor of neurodegeneration. Cell 173, 1073–1081. 10.1016/j.cell.2018.05.003 29775591

[B25] Del Fresno C. Cueto F. J. Sancho D. (2020). Sensing tissue damage by myeloid C-Type lectin receptors. Curr. Top. Microbiol. Immunol. 429, 117–145. 10.1007/82_2019_194 31974758

[B26] Deng S. Sherchan P. Jin P. Huang L. Travis Z. Zhang J. H.. (2020). Recombinant CCL17 enhances hematoma resolution and activation of CCR4/ERK/Nrf2/CD163 signaling pathway after intracerebral hemorrhage in mice. Neurotherapeutics 17, 1940–1953. 10.1007/s13311-020-00908-4 32783091 PMC7851239

[B27] Dong X. Q. Yu W. H. Hu Y. Y. Zhang Z. Y. Huang M. (2011). Oxymatrine reduces neuronal cell apoptosis by inhibiting Toll-like receptor 4/nuclear factor kappa-B-dependent inflammatory responses in traumatic rat brain injury. Inflamm. Res. 60, 533–539. 10.1007/s00011-010-0300-7 21190123

[B28] Drouin M. Saenz J. Chiffoleau E. (2020). C-Type lectin-like receptors: head or tail in cell death immunity. Front. Immunol. 11:251. 10.3389/fimmu.2020.00251 32133013 PMC7040094

[B29] Duan X. C. Wang W. Feng D. X. Yin J. Zuo G. Chen D. D.. (2017). Roles of autophagy and endoplasmic reticulum stress in intracerebral hemorrhage-induced secondary brain injury in rats. CNS Neurosci. Ther. 23, 554–566. 10.1111/cns.12703 28544790 PMC6492729

[B30] Eldahshan W. Fagan S. C. Ergul A. (2019). Inflammation within the neurovascular unit: focus on microglia for stroke injury and recovery. Pharmacol. Res. 147:104349. 10.1016/j.phrs.2019.104349 31315064 PMC6954670

[B31] Fang H. Chen J. Lin S. Wang P. Wang Y. Xiong X.. (2014). CD36-mediated hematoma absorption following intracerebral hemorrhage: negative regulation by TLR4 signaling. J. Immunol. 192, 5984–5992. 10.4049/jimmunol.1400054 24808360 PMC4049082

[B33] Fang Y. Gao S. Wang X. Cao Y. Lu J. Chen S.. (2020). Programmed cell deaths and potential crosstalk with blood-brain barrier dysfunction after hemorrhagic stroke. Front. Cell Neurosci. 14:68. 10.3389/fncel.2020.00068 32317935 PMC7146617

[B34] Fang Y. Tian Y. Huang Q. Wan Y. Xu L. Wang W.. (2019). Deficiency of TREK-1 potassium channel exacerbates blood-brain barrier damage and neuroinflammation after intracerebral hemorrhage in mice. J. Neuroinflammation 16:96. 10.1186/s12974-019-1485-5 31072336 PMC6506965

[B32] Fang H. Wang P. F. Zhou Y. Wang Y. C. Yang Q. W. (2013). Toll-like receptor 4 signaling in intracerebral hemorrhage-induced inflammation and injury. J. Neuroinflammation 10:27. 10.1186/1742-2094-10-27 23414417 PMC3598479

[B35] Florczak-Rzepka M. Grond-Ginsbach C. Montaner J. Steiner T. (2012). Matrix metalloproteinases in human spontaneous intracerebral hemorrhage: an update. Cerebrovasc. Dis. 34, 249–262. 10.1159/000341686 23052179

[B36] Friedman B. A. Srinivasan K. Ayalon G. Meilandt W. J. Lin H. Huntley M. A.. (2018). Diverse brain myeloid expression profiles reveal distinct microglial activation states and aspects of Alzheimer’s disease not evident in mouse models. Cell Rep. 22, 832–847. 10.1016/j.celrep.2017.12.066 29346778

[B37] Gao T. Jernigan J. Raza S. A. Dammer E. B. Xiao H. Seyfried N. T.. (2019). Transcriptional regulation of homeostatic and disease-associated-microglial genes by IRF1, LXRβ and CEBPα. Glia 67, 1958–1975. 10.1002/glia.23678 31301160 PMC7190149

[B38] Garcia-Revilla J. Alonso-Bellido I. M. Burguillos M. A. Herrera A. J. Espinosa-Oliva A. M. Ruiz R.. (2019). Reformulating pro-oxidant microglia in neurodegeneration. J. Clin. Med. 8:1719. 10.3390/jcm8101719 31627485 PMC6832973

[B39] Gong W. Zheng T. Guo K. Fang M. Xie H. Li W.. (2020). Mincle/Syk signalling promotes intestinal mucosal inflammation through induction of macrophage pyroptosis in Crohn’s disease. J. Crohn’s and Colitis 14, 1734–1747. 10.1093/ecco-jcc/jjaa088 32333776

[B40] Gosselin D. Skola D. Coufal N. G. Holtman I. R. Schlachetzki J. C. M. Sajti E.. (2017). An environment-dependent transcriptional network specifies human microglia identity. Science 356:eaal3222. 10.1126/science.aal3222 28546318 PMC5858585

[B41] Hammond T. R. Dufort C. Dissing-Olesen L. Giera S. Young A. Wysoker A.. (2019). Single-cell RNA sequencing of microglia throughout the mouse lifespan and in the injured brain reveals complex cell-state changes. Immunity 50, 253–271.e6. 10.1016/j.immuni.2018.11.004 30471926 PMC6655561

[B42] Hatakeyama T. Okauchi M. Hua Y. Keep R. F. Xi G. (2013). Deferoxamine reduces neuronal death and hematoma lysis after intracerebral hemorrhage in aged rats. Transl. Stroke Res. 4, 546–553. 10.1007/s12975-013-0270-5 24187595 PMC3810989

[B43] He X. Huang Y. Liu Y. Zhang X. Yue P. Ma X.. (2022). BAY613606 attenuates neuroinflammation and neurofunctional damage by inhibiting microglial Mincle/Syk signaling response after traumatic brain injury. Int. J. Mol. Med. 49:5. 10.3892/ijmm.2021.5060 34751408 PMC8612304

[B44] He Y. Xu L. Li B. Guo Z. N. Hu Q. Guo Z.. (2015). Macrophage-inducible C-Type lectin/spleen tyrosine kinase signaling pathway contributes to neuroinflammation after subarachnoid hemorrhage in rats. Stroke 46, 2277–2286. 10.1161/STROKEAHA.115.010088 26138128 PMC4519357

[B45] Hemphill J. C. 3rd Greenberg, S. M. Anderson C. S. Becker K. Bendok B. R. Cushman M.. (2015). Guidelines for the management of spontaneous intracerebral hemorrhage: a guideline for healthcare professionals from the american heart association/american stroke association. Stroke 46, 2032–2060. 10.1161/STR.0000000000000069 26022637

[B47] Hu Y. Y. Huang M. Dong X. Q. Xu Q. P. Yu W. H. Zhang Z. Y. (2011). Ginkgolide B reduces neuronal cell apoptosis in the hemorrhagic rat brain: possible involvement of Toll-like receptor 4/nuclear factor-kappa B pathway. J. Ethnopharmacol. 137, 1462–1468. 10.1016/j.jep.2011.08.034 21878382

[B46] Hu S. Hua Y. Keep R. F. Feng H. Xi G. (2019). Deferoxamine therapy reduces brain hemin accumulation after intracerebral hemorrhage in piglets. Exp. Neurol. 318, 244–250. 10.1016/j.expneurol.2019.05.003 31078524 PMC6588480

[B48] Jiang G. L. Yang X. L. Zhou H. J. Long J. Liu B. Zhang L. M.. (2021). cGAS knockdown promotes microglial M2 polarization to alleviate neuroinflammation by inhibiting cGAS-STING signaling pathway in cerebral ischemic stroke. Brain Res. Bull. 171, 183–195. 10.1016/j.brainresbull.2021.03.010 33745949

[B49] Jin W. N. Shi S. X. Li Z. Li M. Wood K. Gonzales R. J.. (2017). Depletion of microglia exacerbates postischemic inflammation and brain injury. J. Cereb. Blood Flow. Metab. 37, 2224–2236. 10.1177/0271678X17694185 28273719 PMC5444553

[B50] Jing C. Bian L. Wang M. Keep R. F. Xi G. Hua Y.. (2019). Enhancement of hematoma clearance with CD47 blocking antibody in experimental intracerebral hemorrhage. Stroke 50, 1539–1547. 10.1161/STROKEAHA.118.024578 31084334 PMC6538472

[B51] Keren-Shaul H. Spinrad A. Weiner A. Matcovitch-Natan O. Dvir-Szternfeld R. Ulland T. K.. (2017). A unique microglia type associated with restricting development of Alzheimer’s disease. Cell 169, 1276–1290.e17. 10.1016/j.cell.2017.05.018 28602351

[B52] Kim J. W. Roh Y. S. Jeong H. Yi H. K. Lee M. H. Lim C. W.. (2018). Spliceosome-associated protein 130 exacerbates alcohol-induced liver injury by inducing NLRP3 inflammasome-mediated IL-1β in mice. Am. J. Pathol. 188, 967–980. 10.1016/j.ajpath.2017.12.010 29355515

[B53] Klebe D. McBride D. Flores J. J. Zhang J. H. Tang J. (2015). Modulating the immune response towards a neuroregenerative peri-injury milieu after cerebral hemorrhage. J. Neuroimmune Pharmacol. 10, 576–586. 10.1007/s11481-015-9613-1 25946986 PMC4636976

[B54] Lan X. Han X. Li Q. Yang Q. W. Wang J. (2017). Modulators of microglial activation and polarization after intracerebral haemorrhage. Nat. Rev. Neurol. 13, 420–433. 10.1038/nrneurol.2017.69 28524175 PMC5575938

[B55] Lassmann H. (2020). Pathology of inflammatory diseases of the nervous system: human disease versus animal models. Glia 68, 830–844. 10.1002/glia.23726 31605512 PMC7065008

[B56] Lattanzi S. Di Napoli M. Ricci S. Divani A. A. (2020). Matrix metalloproteinases in acute intracerebral hemorrhage. Neurotherapeutics 17, 484–496. 10.1007/s13311-020-00839-0 31975152 PMC7283398

[B57] Lei C. Tan Y. Ni D. Peng J. Yi G. (2022). cGAS-STING signaling in ischemic diseases. Clin. Chim. Acta 531, 177–182. 10.1016/j.cca.2022.04.003 35398249

[B62] Li Y. Dong Y. Ran Y. Zhang Y. Wu B. Xie J.. (2021). Three-dimensional cultured mesenchymal stem cells enhance repair of ischemic stroke through inhibition of microglia. Stem Cell Res. Ther. 12:358. 10.1186/s13287-021-02416-4 34154653 PMC8218508

[B59] Li Q. Lan X. Han X. Durham F. Wan J. Weiland A.. (2021). Microglia-derived interleukin-10 accelerates post-intracerebral hemorrhage hematoma clearance by regulating CD36. Brain Behav. Immun. 94, 437–457. 10.1016/j.bbi.2021.02.001 33588074 PMC8058329

[B64] Li Z. Liu Y. Wei R. Khan S. Zhang R. Zhang Y.. (2022). Iron neurotoxicity and protection by deferoxamine in intracerebral hemorrhage. Front. Mol. Neurosci. 15:927334. 10.3389/fnmol.2022.927334 35782383 PMC9245523

[B61] Li R. Liu Z. Wu X. Yu Z. Zhao S. Tang X. (2019). Lithium chloride promoted hematoma resolution after intracerebral hemorrhage through GSK-3β-mediated pathways-dependent microglia phagocytosis and M2-phenotype differentiation, angiogenesis and neurogenesis in a rat model. Brain Res. Bull. 152, 117–127. 10.1016/j.brainresbull.2019.07.019 31325596

[B60] Li Q. Wan J. Lan X. Han X. Wang Z. Wang J. (2017). Neuroprotection of brain-permeable iron chelator VK-28 against intracerebral hemorrhage in mice. J. Cereb. Blood Flow. Metab. 37, 3110–3123. 10.1177/0271678X17709186 28534662 PMC5584702

[B58] Li M. Xia M. Chen W. Wang J. Yin Y. Guo C.. (2020). Lithium treatment mitigates white matter injury after intracerebral hemorrhage through brain-derived neurotrophic factor signaling in mice. Transl. Res. 217, 61–74. 10.1016/j.trsl.2019.12.006 31951826

[B63] Li Y. Zhu Z. Y. Lu B. W. Huang T. T. Zhang Y. M. Zhou N. Y.. (2019). Rosiglitazone ameliorates tissue plasminogen activator-induced brain hemorrhage after stroke. CNS Neurosci. Ther. 25, 1343–1352. 10.1111/cns.13260 31756041 PMC6887660

[B65] Liang H. Guan D. Gao A. Yin Y. Jing M. Yang L.. (2014). Human amniotic epithelial stem cells inhibit microglia activation through downregulation of tumor necrosis factor-α, interleukin-1β and matrix metalloproteinase-12 *in vitro* and in a rat model of intracerebral hemorrhage. Cytotherapy 16, 523–534. 10.1016/j.jcyt.2013.11.007 24424266

[B69] Liu X. Chen X. Zhu Y. Wang K. Wang Y. (2017). Effect of magnolol on cerebral injury and blood brain barrier dysfunction induced by ischemia-reperfusion *in vivo* and *in vitro*. Metab. Brain Dis. 32, 1109–1118. 10.1007/s11011-017-0004-6 28378105

[B68] Liu X. Y. Dai X. H. Zou W. Yu X. P. Teng W. Wang Y.. (2018). Acupuncture through Baihui (DU20) to Qubin (GB7) mitigates neurological impairment after intracerebral hemorrhage. Neural Regen. Res. 13, 1425–1432. 10.4103/1673-5374.235298 30106055 PMC6108213

[B70] Liu Z. Li R. Jiang C. Zhao S. Li W. Tang X. (2018). The neuroprotective effect of lithium chloride on cognitive impairment through glycogen synthase kinase-3β inhibition in intracerebral hemorrhage rats. Eur. J. Pharmacol. 840, 50–59. 10.1016/j.ejphar.2018.10.019 30336136

[B66] Liu D. L. Zhao L. X. Zhang S. Du J. R. (2016). Peroxiredoxin 1-mediated activation of TLR4/NF-kappaB pathway contributes to neuroinflammatory injury in intracerebral hemorrhage. Int. Immunopharmacol. 41, 82–89. 10.1016/j.intimp.2016.10.025 27821296

[B67] Liu J. Zhu Z. Leung G. K. (2022). Erythrophagocytosis by Microglia/Macrophage in intracerebral hemorrhage: from mechanisms to translation. Front. Cell Neurosci. 16:818602. 10.3389/fncel.2022.818602 35237132 PMC8882619

[B71] Luo Y. Yin W. Signore A. P. Zhang F. Hong Z. Wang S.. (2006). Neuroprotection against focal ischemic brain injury by the peroxisome proliferator-activated receptor-γ agonist rosiglitazone. J. Neurochem. 97, 435–448. 10.1111/j.1471-4159.2006.03758.x 16539667

[B72] Lv Y. N. Ou-Yang A. J. Fu L. S. (2017). MicroRNA-27a negatively modulates the inflammatory response in lipopolysaccharide-stimulated microglia by targeting TLR4 and IRAK4. Cell Mol. Neurobiol. 37, 195–210. 10.1007/s10571-016-0361-4 26971344 PMC11482108

[B73] Masuda T. Sankowski R. Staszewski O. Bottcher C. Amann L. Sagar et al. (2019). Spatial and temporal heterogeneity of mouse and human microglia at single-cell resolution. Nature 566, 388–392. 10.1038/s41586-019-0924-x 30760929

[B74] Mecca C. Giambanco I. Donato R. Arcuri C. (2018). Microglia and aging: the role of the TREM2-DAP12 and CX3CL1-CX3CR1 axes. Int. J. Mol. Sci. 19:318. 10.3390/ijms19010318 29361745 PMC5796261

[B75] Miao H. Li R. Han C. Lu X. Zhang H. (2018). Minocycline promotes posthemorrhagic neurogenesis via M2 microglia polarization via upregulation of the TrkB/BDNF pathway in rats. J. Neurophysiol. 120, 1307–1317. 10.1152/jn.00234.2018 29790836

[B76] Ochocka N. Segit P. Walentynowicz K. A. Wojnicki K. Cyranowski S. Swatler J.. (2021). Single-cell RNA sequencing reveals functional heterogeneity of glioma-associated brain macrophages. Nat. Commun. 12:1151. 10.1038/s41467-021-21407-w 33608526 PMC7895824

[B77] Ohnishi M. Katsuki H. Fujimoto S. Takagi M. Kume T. Akaike A. (2007). Involvement of thrombin and mitogen-activated protein kinase pathways in hemorrhagic brain injury. Exp. Neurol. 206, 43–52. 10.1016/j.expneurol.2007.03.030 17498698

[B78] Olah M. Menon V. Habib N. Taga M. F. Ma Y. Yung C. J.. (2020). Single cell RNA sequencing of human microglia uncovers a subset associated with Alzheimer’s disease. Nat. Commun. 11:6129. 10.1038/s41467-020-19737-2 33257666 PMC7704703

[B79] Ozaki E. Delaney C. Campbell M. Doyle S. L. (2022). Minocycline suppresses disease-associated microglia (DAM) in a model of photoreceptor cell degeneration. Exp. Eye Res. 217:108953. 10.1016/j.exer.2022.108953 35090890

[B80] Prinz M. Erny D. Hagemeyer N. (2017). Ontogeny and homeostasis of CNS myeloid cells. Nat. Immunol. 18, 385–392. 10.1038/ni.3703 28323268

[B81] Prinz M. Jung S. Priller J. (2019). Microglia biology: one century of evolving concepts. Cell 179, 292–311. 10.1016/j.cell.2019.08.053 31585077

[B82] Rangaraju S. Dammer E. B. Raza S. A. Rathakrishnan P. Xiao H. Gao T.. (2018). Identification and therapeutic modulation of a pro-inflammatory subset of disease-associated-microglia in Alzheimer’s disease. Mol. Neurodegener. 13:24. 10.1186/s13024-018-0254-8 29784049 PMC5963076

[B83] Ransohoff R. M. (2016). A polarizing question: do M1 and M2 microglia exist? Nat. Neurosci. 19, 987–991. 10.1038/nn.4338 27459405

[B84] Ronaldson P. T. Davis T. P. (2020). Regulation of blood-brain barrier integrity by microglia in health and disease: a therapeutic opportunity. J. Cereb. Blood Flow Metab. 40, S6–S24. 10.1177/0271678X20951995 32928017 PMC7687032

[B85] Saito M. Saito M. Das B. C. (2019). Involvement of AMP-activated protein kinase in neuroinflammation and neurodegeneration in the adult and developing brain. Int. J. Dev. Neurosci. 77, 48–59. 10.1016/j.ijdevneu.2019.01.007 30707928 PMC6663660

[B86] Sansing L. H. Harris T. H. Welsh F. A. Kasner S. E. Hunter C. A. Kariko K. (2011). Toll-like receptor 4 contributes to poor outcome after intracerebral hemorrhage. Ann. Neurol. 70, 646–656. 10.1002/ana.22528 22028224 PMC3671585

[B87] Sekerdag E. Solaroglu I. Gursoy-Ozdemir Y. (2018). Cell death mechanisms in stroke and novel molecular and cellular treatment options. Curr. Neuropharmacol. 16, 1396–1415. 10.2174/1570159X16666180302115544 29512465 PMC6251049

[B88] Shang Y. Dai S. Chen X. Wen W. Liu X. (2019). MicroRNA-93 regulates the neurological function, cerebral edema and neuronal apoptosis of rats with intracerebral hemorrhage through TLR4/NF-κB signaling pathway. Cell Cycle 18, 3160–3176. 10.1080/15384101.2019.1670509 31559899 PMC6816398

[B89] Sharma M. Rajendrarao S. Shahani N. Ramirez-Jarquin U. N. Subramaniam S. (2020). Cyclic GMP-AMP synthase promotes the inflammatory and autophagy responses in Huntington disease. Proc. Natl. Acad. Sci. U S A 117, 15989–15999. 10.1073/pnas.2002144117 32581130 PMC7354937

[B92] Shi S. X. Li Y. J. Shi K. Wood K. Ducruet A. F. Liu Q. (2020). IL (Interleukin)-15 bridges astrocyte-microglia crosstalk and exacerbates brain injury following intracerebral hemorrhage. Stroke 51, 967–974. 10.1161/STROKEAHA.119.028638 32019481

[B90] Shi H. Wang J. Wang J. Huang Z. Yang Z. (2018). IL-17A induces autophagy and promotes microglial neuroinflammation through ATG5 and ATG7 in intracerebral hemorrhage. J. Neuroimmunol. 323, 143–151. 10.1016/j.jneuroim.2017.07.015 28778418

[B91] Shi J. Yang Y. Yin N. Liu C. Zhao Y. Cheng H.. (2022). Engineering CXCL12 biomimetic decoy-integrated versatile immunosuppressive nanoparticle for ischemic stroke therapy with management of overactivated brain immune microenvironment. Small Methods 6:e2101158. 10.1002/smtd.202101158 35041278

[B93] Su E. J. Cao C. Fredriksson L. Nilsson I. Stefanitsch C. Stevenson T. K.. (2017). Microglial-mediated PDGF-CC activation increases cerebrovascular permeability during ischemic stroke. Acta Neuropathol. 134, 585–604. 10.1007/s00401-017-1749-z 28725968 PMC5587628

[B94] Tan X. Yang Y. Xu J. Zhang P. Deng R. Mao Y.. (2020). Luteolin exerts neuroprotection via modulation of the p62/Keap1/Nrf2 pathway in intracerebral hemorrhage. Front. Pharmacol. 10:1551. 10.3389/fphar.2019.01551 32038239 PMC6985769

[B95] Taylor R. A. Chang C. F. Goods B. A. Hammond M. D. Mac Grory B. Ai Y.. (2017). TGF-β1 modulates microglial phenotype and promotes recovery after intracerebral hemorrhage. J. Clin. Invest. 127, 280–292. 10.1172/JCI88647 27893460 PMC5199690

[B96] Tschoe C. Bushnell C. D. Duncan P. W. Alexander-Miller M. A. Wolfe S. Q. (2020). Neuroinflammation after intracerebral hemorrhage and potential therapeutic targets. J. Stroke 22, 29–46. 10.5853/jos.2019.02236 32027790 PMC7005353

[B97] Vainchtein I. D. Molofsky A. V. (2020). Astrocytes and microglia: in sickness and in health. Trends Neurosci. 43, 144–154. 10.1016/j.tins.2020.01.003 32044129 PMC7472912

[B98] Vinukonda G. Liao Y. Hu F. Ivanova L. Purohit D. Finkel D. A.. (2019). Human cord blood-derived unrestricted somatic stem cell infusion improves neurobehavioral outcome in a rabbit model of intraventricular hemorrhage. Stem Cells Transl. Med. 8, 1157–1169. 10.1002/sctm.19-0082 31322326 PMC6811700

[B99] Voet S. Prinz M. van Loo G. (2019). Microglia in central nervous system inflammation and multiple sclerosis pathology. Trends Mol. Med. 25, 112–123. 10.1016/j.molmed.2018.11.005 30578090

[B100] Wan S. Cheng Y. Jin H. Guo D. Hua Y. Keep R. F.. (2016). Microglia activation and polarization after intracerebral hemorrhage in mice: the role of protease-activated receptor-1. Transl. Stroke Res. 7, 478–487. 10.1007/s12975-016-0472-8 27206851 PMC5065741

[B103] Wang J. (2010). Preclinical and clinical research on inflammation after intracerebral hemorrhage. Prog. Neurobiol. 92, 463–477. 10.1016/j.pneurobio.2010.08.001 20713126 PMC2991407

[B109] Wang Y. Chen Q. Tan Q. Feng Z. He Z. Tang J.. (2018). Simvastatin accelerates hematoma resolution after intracerebral hemorrhage in a PPARγ-dependent manner. Neuropharmacology 128, 244–254. 10.1016/j.neuropharm.2017.10.021 29054366

[B106] Wang M. Hua Y. Keep R. F. Wan S. Novakovic N. Xi G.. (2019a). Complement inhibition attenuates early erythrolysis in the hematoma and brain injury in aged rats. Stroke 50, 1859–1868. 10.1161/STROKEAHA.119.025170 31177985 PMC6591097

[B105] Wang M. Cheng L. Chen Z. L. Mungur R. Xu S. H. Wu J.. (2019b). Hyperbaric oxygen preconditioning attenuates brain injury after intracerebral hemorrhage by regulating microglia polarization in rats. CNS Neurosci. Ther. 25, 1126–1133. 10.1111/cns.13208 31411803 PMC6776759

[B101] Wang G. Shi Y. Jiang X. Leak R. K. Hu X. Wu Y.. (2015). HDAC inhibition prevents white matter injury by modulating microglia/macrophage polarization through the GSK3β/PTEN/Akt axis. Proc. Natl. Acad. Sci. U S A 112, 2853–2858. 10.1073/pnas.1501441112 25691750 PMC4352818

[B108] Wang Y. C. Wang P. F. Fang H. Chen J. Xiong X. Y. Yang Q. W. (2013). Toll-like receptor 4 antagonist attenuates intracerebral hemorrhage-induced brain injury. Stroke 44, 2545–2552. 10.1161/STROKEAHA.113.001038 23839500

[B102] Wang G. Wang L. Sun X. G. Tang J. (2018). Haematoma scavenging in intracerebral haemorrhage: from mechanisms to the clinic. J. Cell. Mol. Med. 22, 768–777. 10.1111/jcmm.13441 29278306 PMC5783832

[B104] Wang J. Xu Z. Chen X. Li Y. Chen C. Wang C.. (2018). MicroRNA-182-5p attenuates cerebral ischemia-reperfusion injury by targeting Toll-like receptor 4. Biochem. Biophys. Res. Commun. 505, 677–684. 10.1016/j.bbrc.2018.09.165 30292407

[B110] Wang Z. Yuan B. Fu F. Huang S. Yang Z. (2017). Hemoglobin enhances miRNA-144 expression and autophagic activation mediated inflammation of microglia via mTOR pathway. Sci. Rep. 7:11861. 10.1038/s41598-017-12067-2 28928406 PMC5605685

[B107] Wang R. Zhu Y. Liu Z. Chang L. Bai X. Kang L.. (2021). Neutrophil extracellular traps promote tPA-induced brain hemorrhage via cGAS in mice with stroke. Blood 138, 91–103. 10.1182/blood.2020008913 33881503 PMC8288643

[B111] Wasserman J. K. Schlichter L. C. (2007). Minocycline protects the blood-brain barrier and reduces edema following intracerebral hemorrhage in the rat. Exp. Neurol. 207, 227–237. 10.1016/j.expneurol.2007.06.025 17698063

[B112] Wasserman J. K. Zhu X. Schlichter L. C. (2007). Evolution of the inflammatory response in the brain following intracerebral hemorrhage and effects of delayed minocycline treatment. Brain Res. 1180, 140–154. 10.1016/j.brainres.2007.08.058 17919462

[B113] Wells J. E. Biernaskie J. Szymanska A. Larsen P. H. Yong V. W. Corbett D. (2005). Matrix metalloproteinase (MMP)-12 expression has a negative impact on sensorimotor function following intracerebral haemorrhage in mice. Eur. J. Neurosci. 21, 187–196. 10.1111/j.1460-9568.2004.03829.x 15654856

[B114] Wolf S. A. Boddeke H. W. Kettenmann H. (2017). Microglia in physiology and disease. Annu. Rev. Physiol. 79, 619–643. 10.1146/annurev-physiol-022516-034406 27959620

[B115] Wu C. H. Shyue S. K. Hung T. H. Wen S. Lin C. C. Chang C. F.. (2017). Genetic deletion or pharmacological inhibition of soluble epoxide hydrolase reduces brain damage and attenuates neuroinflammation after intracerebral hemorrhage. J. Neuroinflammation 14:230. 10.1186/s12974-017-1005-4 29178914 PMC5702198

[B116] Wu H. Wu T. Xu X. Wang J. Wang J. (2011). Iron toxicity in mice with collagenase-induced intracerebral hemorrhage. J. Cereb. Blood Flow Metab. 31, 1243–1250. 10.1038/jcbfm.2010.209 21102602 PMC3099628

[B117] Xiao H. Chen H. Jiang R. Zhang L. Wang L. Gan H.. (2020). NLRP6 contributes to inflammation and brain injury following intracerebral haemorrhage by activating autophagy. J. Mol. Med. (Berl) 98, 1319–1331. 10.1007/s00109-020-01962-3 32783081

[B122] Xu Y. J. Au N. P. B. Ma C. H. E. (2022). Functional and phenotypic diversity of microglia: implication for microglia-based therapies for Alzheimer’s disease. Front. Aging Neurosci. 14:896852. 10.3389/fnagi.2022.896852 35693341 PMC9178186

[B118] Xu H. Cao J. Xu J. Li H. Shen H. Li X.. (2019). GATA-4 regulates neuronal apoptosis after intracerebral hemorrhage via the NF-κB/Bax/Caspase-3 pathway both *in vivo* and *in vitro*. Exp. Neurol. 315, 21–31. 10.1016/j.expneurol.2019.01.018 30710529

[B119] Xu J. Chen Z. Yu F. Liu H. Ma C. Xie D.. (2020). IL-4/STAT6 signaling facilitates innate hematoma resolution and neurological recovery after hemorrhagic stroke in mice. Proc. Natl. Acad. Sci. U S A 117, 32679–32690. 10.1073/pnas.2018497117 33293423 PMC7768771

[B121] Xu R. Wang S. Li W. Liu Z. Tang J. Tang X. (2017). Activation of peroxisome proliferator-activated receptor-γ by a 12/15-lipoxygenase product of arachidonic acid: a possible neuroprotective effect in the brain after experimental intracerebral hemorrhage. J. Neurosurg. 127, 522–531. 10.3171/2016.7.JNS1668 27739938

[B120] Xu N. Zhang Y. Doycheva D. M. Ding Y. Zhang Y. Tang J.. (2018). Adiponectin attenuates neuronal apoptosis induced by hypoxia-ischemia via the activation of AdipoR1/APPL1/LKB1/AMPK pathway in neonatal rats. Neuropharmacology 133, 415–428. 10.1016/j.neuropharm.2018.02.024 29486166

[B123] Yang G. Fan X. Mazhar M. Yang S. Xu H. Dechsupa N.. (2022). Mesenchymal stem cell application and its therapeutic mechanisms in intracerebral hemorrhage. Front. Cell Neurosci. 16:898497. 10.3389/fncel.2022.898497 35769327 PMC9234141

[B126] Yang Z. Jiang X. Zhang J. Huang X. Zhang X. Wang J.. (2018). Let-7a promotes microglia M2 polarization by targeting CKIP-1 following ICH. Immunol. Lett. 202, 1–7. 10.1016/j.imlet.2018.07.007 30053453

[B127] Yang Z. Liu B. Zhong L. Shen H. Lin C. Lin L.. (2015). Toll-like receptor-4-mediated autophagy contributes to microglial activation and inflammatory injury in mouse models of intracerebral haemorrhage. Neuropathol. Appl. Neurobiol. 41, e95–e106. 10.1111/nan.12177 25185720

[B125] Yang X. Ren H. Wood K. Li M. Qiu S. Shi F. D.. (2018). Depletion of microglia augments the dopaminergic neurotoxicity of MPTP. FASEB J. 32, 3336–3345. 10.1096/fj.201700833RR 29401614 PMC5956250

[B124] Yang L. Tang J. Chen Q. Jiang B. Zhang B. Tao Y.. (2015). Hyperbaric oxygen preconditioning attenuates neuroinflammation after intracerebral hemorrhage in rats by regulating microglia characteristics. Brain Res. 1627, 21–30. 10.1016/j.brainres.2015.08.011 26301824

[B129] Yang Z. Zhao T. Zou Y. Zhang J. H. Feng H. (2014a). Curcumin inhibits microglia inflammation and confers neuroprotection in intracerebral hemorrhage. Immunol. Lett. 160, 89–95. 10.1016/j.imlet.2014.03.005 24680995

[B128] Yang Z. Yu A. Liu Y. Shen H. Lin C. Lin L.. (2014b). Regulatory T cells inhibit microglia activation and protect against inflammatory injury in intracerebral hemorrhage. Int. Immunopharmacol. 22, 522–525. 10.1016/j.intimp.2014.06.037 25000335

[B130] Yu A. Duan H. Zhang T. Pan Y. Kou Z. Zhang X.. (2016). IL-17A promotes microglial activation and neuroinflammation in mouse models of intracerebral haemorrhage. Mol. Immunol. 73, 151–157. 10.1016/j.molimm.2016.04.003 27107665

[B132] Yu A. Zhang T. Zhong W. Duan H. Wang S. Ye P.. (2017a). miRNA-144 induces microglial autophagy and inflammation following intracerebral hemorrhage. Immunol. Lett. 182, 18–23. 10.1016/j.imlet.2017.01.002 28062218

[B131] Yu A. Zhang T. Duan H. Pan Y. Zhang X. Yang G.. (2017b). MiR-124 contributes to M2 polarization of microglia and confers brain inflammatory protection via the C/EBP-α pathway in intracerebral hemorrhage. Immunol. Lett. 182, 1–11. 10.1016/j.imlet.2016.12.003 28025043

[B133] Yuan B. Shen H. Lin L. Su T. Zhong L. Yang Z. (2017). Autophagy promotes microglia activation through beclin-1-atg5 pathway in intracerebral hemorrhage. Mol. Neurobiol. 54, 115–124. 10.1007/s12035-015-9642-z 26732594

[B134] Zhang X. D. Fan Q. Y. Qiu Z. Chen S. (2018). MiR-7 alleviates secondary inflammatory response of microglia caused by cerebral hemorrhage through inhibiting TLR4 expression. Eur. Rev. Med. Pharmacol. Sci. 22, 5597–5604. 10.26355/eurrev_201809_15824 30229834

[B135] Zhang Y. Han B. He Y. Li D. Ma X. Liu Q.. (2017). MicroRNA-132 attenuates neurobehavioral and neuropathological changes associated with intracerebral hemorrhage in mice. Neurochem. Int. 107, 182–190. 10.1016/j.neuint.2016.11.011 27940326

[B136] Zhang Z. Zhang Z. Lu H. Yang Q. Wu H. Wang J.. (2017). Microglial polarization and inflammatory mediators after intracerebral hemorrhage. Mol. Neurobiol. 54, 1874–1886. 10.1007/s12035-016-9785-6 26894396 PMC4991954

[B138] Zhao L. Chen S. Sherchan P. Ding Y. Zhao W. Guo Z.. (2018). Recombinant CTRP9 administration attenuates neuroinflammation via activating adiponectin receptor 1 after intracerebral hemorrhage in mice. J. Neuroinflammation 15:215. 10.1186/s12974-018-1256-8 30060752 PMC6066941

[B141] Zhao X. Grotta J. Gonzales N. Aronowski J. (2009). Hematoma resolution as a therapeutic target: the role of microglia/macrophages. Stroke 40, S92–S94. 10.1161/STROKEAHA.108.533158 19064796

[B137] Zhao F. Hua Y. He Y. Keep R. F. Xi G. (2011). Minocycline-induced attenuation of iron overload and brain injury after experimental intracerebral hemorrhage. Stroke 42, 3587–3593. 10.1161/STROKEAHA.111.623926 21998050 PMC3226873

[B140] Zhao W. Kong F. Gong X. Guo Z. Zhao L. Wang S. (2021). Activation of AdipoR1 with rCTRP9 preserves BBB integrity through the APPL1/AMPK/Nrf2 signaling pathway in ICH mice. Oxid. Med. Cell. Longev. 2021:2801263. 10.1155/2021/2801263 34925690 PMC8674037

[B139] Zhao S. Liu Z. Yu Z. Wu X. Li R. Tang X. (2019). BIO alleviates inflammation through inhibition of GSK-3β in a rat model of intracerebral hemorrhage. J. Neurosurg. 1–9. 10.3171/2019.4.JNS183501. [Online ahead of print]. 31226691

[B142] Zhao X. Sun G. Zhang J. Strong R. Song W. Gonzales N.. (2007). Hematoma resolution as a target for intracerebral hemorrhage treatment: role for peroxisome proliferator-activated receptor γ in microglia/macrophages. Ann. Neurol. 61, 352–362. 10.1002/ana.21097 17457822

[B143] Zhao Y. Wei Z. Z. Zhang J. Y. Zhang Y. Won S. Sun J.. (2017). GSK-3β inhibition induced neuroprotection, regeneration and functional recovery after intracerebral hemorrhagic stroke. Cell Transplant. 26, 395–407. 10.3727/096368916X694364 28195036 PMC5657706

[B144] Zheng J. Liu Z. Li W. Tang J. Zhang D. Tang X. (2017). Lithium posttreatment confers neuroprotection through glycogen synthase kinase-3β inhibition in intracerebral hemorrhage rats. J. Neurosurg. 127, 716–724. 10.3171/2016.7.JNS152995 27739937

[B146] Zhou Y. Wang Y. Wang J. Anne Stetler R. Yang Q. W. (2014). Inflammation in intracerebral hemorrhage: from mechanisms to clinical translation. Prog. Neurobiol. 115, 25–44. 10.1016/j.pneurobio.2013.11.003 24291544

[B145] Zhou K. Zhong Q. Wang Y. C. Xiong X. Y. Meng Z. Y. Zhao T.. (2017). Regulatory T cells ameliorate intracerebral hemorrhage-induced inflammatory injury by modulating microglia/macrophage polarization through the IL-10/GSK3β/PTEN axis. J. Cereb. Blood Flow Metab. 37, 967–979. 10.1177/0271678X16648712 27174997 PMC5363473

[B147] Zhuang J. Peng Y. Gu C. Chen H. Lin Z. Zhou H.. (2021). Wogonin accelerates hematoma clearance and improves neurological outcome via the PPAR-γ pathway after intracerebral hemorrhage. Transl. Stroke Res. 12, 660–675. 10.1007/s12975-020-00842-9 32918259

[B148] Ziai W. C. (2013). Hematology and inflammatory signaling of intracerebral hemorrhage. Stroke 44, S74–S78. 10.1161/STROKEAHA.111.000662 23709738 PMC12054399

[B149] Zlokovic B. V. (2008). The blood-brain barrier in health and chronic neurodegenerative disorders. Neuron 57, 178–201. 10.1016/j.neuron.2008.01.003 18215617

